# Classification of Parkinson’s Disease in Patch-Based MRI of Substantia Nigra

**DOI:** 10.3390/diagnostics13172827

**Published:** 2023-08-31

**Authors:** Sayyed Shahid Hussain, Xu Degang, Pir Masoom Shah, Saif Ul Islam, Mahmood Alam, Izaz Ahmad Khan, Fuad A. Awwad, Emad A. A. Ismail

**Affiliations:** 1 School of Automation, Central South University, Changsha 410010, China; shahid@csu.edu.cn; 2 Department of Computer Science, Bacha Khan University Charsadda, Charsadda 24540, Pakistan; pirmasoomshah@bkuc.edu.pk (P.M.S.); azaz@bkuc.edu.pk (I.A.K.); 3 School of Computer Science and Engineering, Central South University, Changsha 410010, China; 204708006@csu.edu.cn; 4 Department of Computer Science, Institute of Space Technology, Islamabad 44000, Pakistan; saiflu2004@gmail.com; 5 Department of Quantitative Analysis, College of Business Administration, King Saud University, P.O. Box 71115, Riyadh 11587, Saudi Arabia; fawwad@ksu.edu.sa (F.A.A.); emadali@ksu.edu.sa (E.A.A.I.)

**Keywords:** Parkinson’s disease, convolutional neural networks, MRI

## Abstract

Parkinson’s disease (PD) is a chronic and progressive neurological disease that mostly shakes and compromises the motor system of the human brain. Patients with PD can face resting tremors, loss of balance, bradykinesia, and rigidity problems. Complex patterns of PD, i.e., with relevance to other neurological diseases and minor changes in brain structure, make the diagnosis of this disease a challenge and cause inaccuracy of about 25% in the diagnostics. The research community utilizes different machine learning techniques for diagnosis using handcrafted features. This paper proposes a computer-aided diagnostic system using a convolutional neural network (CNN) to diagnose PD. CNN is one of the most suitable models to extract and learn the essential features of a problem. The dataset is obtained from Parkinson’s Progression Markers Initiative (PPMI), which provides different datasets (benchmarks), such as T2-weighted MRI for PD and other healthy controls (HC). The mid slices are collected from each MRI. Further, these slices are registered for alignment. Since the PD can be found in substantia nigra (i.e., the midbrain), the midbrain region of the registered T2-weighted MRI slice is selected using the freehand region of interest technique with a 33 × 33 sized window. Several experiments have been carried out to ensure the validity of the CNN. The standard measures, such as accuracy, sensitivity, specificity, and area under the curve, are used to evaluate the proposed system. The evaluation results show that CNN provides better accuracy than machine learning techniques, such as naive Bayes, decision tree, support vector machine, and artificial neural network.

## 1. Introduction

Parkinson’s disease (PD) is one of the brain diseases that occur due to disorder in the neurological system of the brain. The thalamus is a region in the human brain that contains neurons and has an important role in transmitting sensory information to the brain. Another region of the human brain is the substantia nigra, which contains dopaminergic neurons. Dopamine, a neurotransmitter essential for motor coordination and control, is produced and released by these neurons [1]. Dopamine provides signals to the brain and other parts of the body related to movement and coordination. During Parkinson’s disease, dopamine chemical generation decreases and causes neuron death [2]. Parkinson’s disease symptoms include shakes, slowness in muscle movement, stiffness, imbalance, or postural instability. There are some other symptoms as well, such as slowness in thinking, voice disorder, fatigue, anxiety, and depression. Sleep may also become disturbed and concentration may be lost [3]. There is no medical lab or medical test to diagnose this disease [4]. Traditionally, medical experts have used past records and neurological investigations. However, this approach is not that accurate because of many reasons and similar neurodegeneration diseases. It is difficult to diagnose after much loss of dopamine chemicals. The correct detection of PD is very important. If a patient is diagnosed as healthy, with time, this disease becomes worse, which is difficult to control. Machine learning is widely used in many medical disease diagnoses, like heart disease detection, cancer detection, Alzheimer’s disease detection, and many more. Regarding PD, there are many symptoms that can be present in a Parkinson’s disease patient. These symptoms or features can be age, voice, brain images, etc., in different patterns. So, on the basis of these features, we can classify this disease as PD if a patient has these features or symptoms by using machine learning techniques. In the current era of technology, the trend of making everything automated has been started, which is reaching medical diagnosis as well. Automation can increase the speed and precision of medical diagnosis. Healthcare professionals can gain from the helpful decision making assistance that automated technologies can provide. By employing vast amounts of medical information and data, these technologies can assist physicians in making informed decisions, providing likely diagnoses, and prescribing appropriate tests or treatments. This can help in standardizing diagnostic processes, ensuring consistency in evaluations, and reducing diagnostic variability. These tools can aid in the early identification and prevention of sickness. Automation enables scalability and improved accessibility of medical diagnostics. Different automated and semi-automated systems have been developed for disease classifications [4,5,6,7,8,9]. In the same way, different researchers have attempted to classify PD by using machine-learning-based techniques. Most of these techniques are suport vector machine, neural network, Bayesian learning, decision tree method, etc. In articles [10,11,12], different machine-learning-based approaches that have been applied on people with PD are discussed. A research work in [13] applied the random forest approach on a dataset adopted from ADRC, which contains voice recordings of people with PD and healthy controls. The simulation results showed a 99.25% accuracy rate. However, this technique is not applied on different features and datasets.

### Parkinson’s Classification Based on Machine Learning (ML) and Deep Learning (DL) Techniques

This section is dedicated to recent literature on Parkinson’s classification using different machine learning (ML) and deep learning (DL) techniques. Most of the techniques are fully automated, while some are semi-automated.

A work in [14] proposed a novel intelligent model using DL techniques that analyzes gait information. In order to build deep neural network architecture, a 1D convolutional network is used. The model receives 18-ID signals from foot sensors, which measure vertical ground reaction force (VGRF). The algorithm is tested on Parkinson’s detection and prediction of severity of Parkinson’s. The authors claimed an accuracy of 98.7% achieved by the proposed model.

In [15], the authors introduced an intelligent system that can detect PD from vowels. The features from the vowels are extracted by using singular value decomposition (SVD) and minimum average maximum (MAMa) tree. Further, 50 distinctive features are selected using feature selection techniques. For classification purposes, they used KNN classifier and obtained 92% accuracy.

In [16], they presented a CNN-based model for classification of PD and HC from neuromelanin-sensitive magnetic resonance imaging (NMS-MRI). Neuromelanin-sensitive MRI is a medical imaging technique that allows experts to study the abnormities with detail in substantia nigra pars compacta (SNc). The dataset used in this study comprises the NMS-MRI of 45 subjects in total, where 25 are PD and 35 are HC. The authors claim a superior testing accuracy of 80%.

In [17], the authors proposed a novel intelligent system, where all regions of the brain are covered by a network. Feature vectors are collected from every region of the brain and random forest is used to select relevant features. Lastly, support vector machine is applied in order to combine all the futures along with the ground truth. This model is trained and tested on the Parkinson’s Progression Markers Initiative (PPMI) dataset, including 169 HC and 374 PD subjects. The authors claimed an accuracy of 93%.

The article in [18] proposed a machine-learning-based technique to diagnose Parkinson’s disease by developing a multilayer feed forward neural network (MLFNN). They obtained the dataset from Oxford Parkinson’s datasets, which include the voice measurements of 31 subjects, where 21 of them are PD patients, while rest of the subjects are healthy controls. In total, eight different attributes on the basis of frequency (tremor) are selected. For classification, the k-means algorithm is used. The simulation results showed sensitivity 83.3%, specificity 63.6%, and accuracy 80%.

In [19], another model of PD classification was introduced. The dataset used in this study is adopted from the UCI repository. The swarm optimization technique has been applied for features extraction, while naive Bayes has been applied for classification. The authors claimed 97.5% accuracy.

In [20], the authors used non-motor features for diagnosis purposes of PD. These features are the collection of olfactory loss, sleep behavior disorder, and rapid eye movement (REM). Further, the non-motor features were combined with dopaminergic imaging markers and cerebrospinal fluid measurement features. The dataset used in the experiments was obtained from PPMI, in which 401 were PD subjects while 183 were healthy controls. Boosted tree, SVM, random forest, and Bayes were used for classification purposes. The results showed 96.4% in terms of accuracy with SVM. In the literature, it was studied that non-motor symptoms, including cognitive decline, trouble sleeping, mood problems, and autonomic dysfunction, may show up in the early stages of Parkinson’s disease (PD), even before the appearance of motor symptoms. By considering non-motor traits in addition to motor symptoms, clinical experts can make a more accurate and speedy diagnosis, leading to appropriate treatment and therapy. In addition to Parkinson’s disease, other neurological illnesses can also cause non-motor symptoms. The specific pattern and combination of non-motor symptoms can assist differentiating PD from other disorders to aid in the differential diagnosis process. In PD, non-motor symptoms could manifest before those that are motor.

In [21], the author proposed a novel intelligent model for classifation of PD. This approach is based on GA)-Walvet kernet(WK)-Extreme learning machine (ELM). The neural network was trained by ELM. WK-ELM uses three different parameters, which are adjustable. The ideal values for parameters are calculated with the support of genetic algorithm. The authors obtained a 96% accuracy rate with a dataset taken from the UCI library, which contains voice measurements of 31 subjects, where 23 are PD patients.

In [22], a CNN model, AlexNet, was presented for classifation of PD. The model is trained on 2820 HC and 3296 PD MR images and tested on 705 HC and 824 PD MR images using the transfer learning technique. The PPMI dataset was used in this study. This model achieved 88.9%, 89.30%, and 88.40% results in terms of accuracy, sensitivity, and specificity, respectively.

In [13], the authors performed experiments on Parkinson’s and Alzheimer’s diseases. A fully automated system was introduced based on different intelligence and deep learning algorithms, such as decision tree, random forest, boosted tree, bagging, and MLP. The dataset used in the research was adopted form Alzheimer’s Disease Research Center (ADRC), which contained a total of 890 subjects’ data, where 65% of cases belonged to Alzheimer’s, while 40% were PD subjects. According to this paper, alcohol, genes, and age are the main influencing factors regarding AD and PD. According to the author, accuracy of 99.25% has been achieved on random forest and MLP. A research work in [23] worked on susceptibility weighted imaging (SWI) scan. SWI is a medical imaging technique in MRI. This technique has the capability to visualize the susceptible variations in detail for many issues like blood iron, with the support of contract enhancement. SVM is used for classification of Parkinson’s and Parkinsonisms at an isolated level and obtained an accuracy of 86%. A local dataset is used, having 36 subjects’ records, where 16 were PD patients and 20 were Parkinsonisms.

In [24], the authors worked on three classes of classification regrading PD, progressive supranuclear palsy (PSP), and HC. The advanced stage of PD is PSP; its progression is very high and it is less reactive to medication. The dataset used in this study consists of the MRIs of 84 subjects. The authors applied principal computer analysis (PCA) for feature extraction, while SVM was used as a classifier. Their accuracy is about 88% on an average basis.

In [25], a multimodel on MR images was proposed. In this study, SVM was applied as a classifier. This model obtained the results 86.96 %, 92.59 %, and 78.95% in terms of accuracy, specificity, and sensitivity, respectively. A local dataset was used in this research, which contained a total of 46 subjects, where 19 belonged to PD and 27 belonged to HC.

An author in [26] worked on TRODAT and SPECT images to detect the PD. In this regard, the authors presented an ANN-based model. Striatal and striatum pixel values were obtained from images, and these were then fed to ANN as input. This model obtained an accuracy of 94%.

A comprehensive analysis of prior work is presented in Table 1.

**Figure 1:**
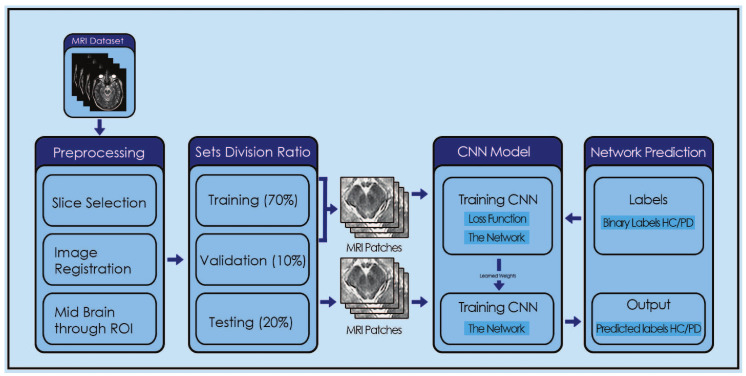
System diagram.

There are different factors involved in PD patients, like olfactory loss, rapid eye disorder, sleep disturbance, postural unbalancing, cerebrospinal fluid, and dopaminergic imaging. There is a need to consider all these features and apply a classification technique that can correctly diagnose people with PD. CNN has shown state-of-the-art accuracy in a number of biomedical image classifications. Recently, Billones, Ciprian D. et al. [35] adjusted the parameters of a VGGNet model for Alzheimer’s detection and succeeded with 91.85% accuracy. Likewise, [36] obtained an accuracy of 93.16% for cerebral microbleeds in MRI. Due to the high accuracy of CNN with MR images, it is applied for PD detection and succeeded in obtaining satisfactory results. The main advantage of the proposed system is that it is a simple convolutional network with limited training parameters; hence, the training time is shorter than state-of-the-art models. A general limitation of the proposed model is that it deals with Parkinson’s disease as a binary classification problem; however,; there are some other diseases closely related to Parkinson’s, such as Parkinsonism, dementia, and Alzheimer’s, etc. It would be good to develop a system that can classify these diseases in a multiclass classification. Figure 1 shows the overall operation of the proposed system.

Regarding the order of the remainder of this research paper, Section 2 covers the materials and methods. The results and experiments of the proposed methods are discussed in Section 3. Section 4 is reserved for the discussion regarding the results. Finally. Section 5 concludes the research and also presents the future work. The main contributions of this paper are four-fold:

1. Achieved the state-of-the-art mean accuracy, sensitivity, specificity, and area under the curve as 96, 96.87, 95.83, and 94.5 percent, respectively.
2. Dealing with limited data, this model was developed in such a manner that reduces the overfitting problem.
3. Low-computational-power GPU was used and obtained satisfactory results as compared to other techniques.
4. Specific patches were extracted from the samples.

## 2. Materials and Methods

### 2.1. Data Acquisition

**Figure 2:**
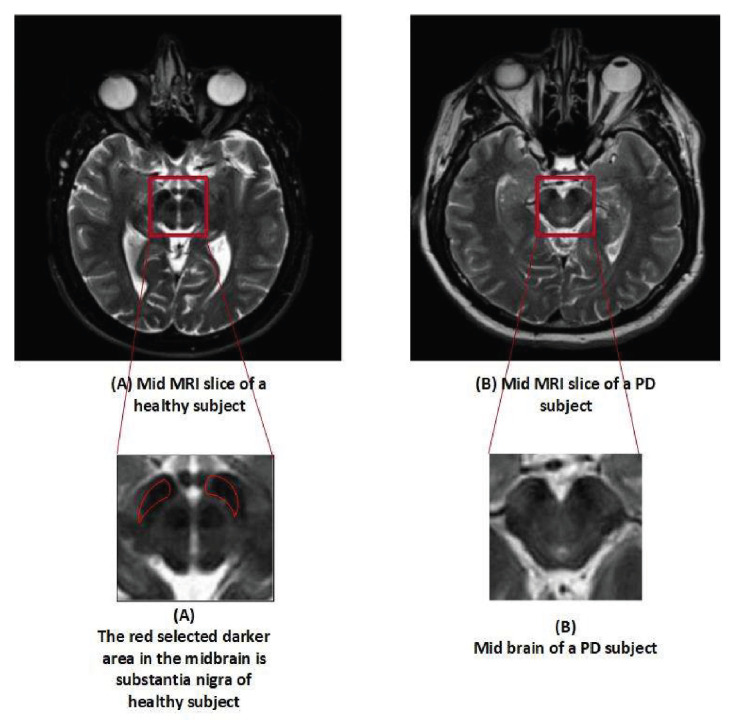
Slices of an MRI scan of an HC and PD patient.

The dataset utilized in this analysis was made available by the PPMI. The PPMI is a multi-study facility with the goal of discovering trustworthy biomarkers and performing an early Parkinson’s disease diagnosis. Additionally, it is the project with the greatest data, which includes a sizable number of clinical, imaging, and biological samples. It is claimed that PPMI offers the largest dataset of its kind, and their samples are known as the benchmark of PD for research purposes across the globe [37]. A total of 500 samples (T2 weighted MR scan) were obtained in Digital Imaging and Communications in Medicine (DICOM) format with the followed parameters, Plane=AXIAL Acquisition Flip Angle = 150.0 degree, Matrix X = 228.0 pixels, Matrix Y = 256.0 pixels, Matrix Z = 54.0, Slice Thickness = 6.0 mm, Pulse Sequence=Spin echo, Pixel Spacing Y = 0.9375 mm, Pixel Spacing X = 0.9375 mm. The data contained 250 numbers of PD and 250 HC samples, balanced data. Dataset is used in such a way that 70% is used for training, 20% for testing, while 10% for validation. The dataset is available on (http://www.ppmi-info.org). Table 2 represents the details of the subject in terms of gender and age, while Figure 2 shows the difference between the MRI scan of a healthy subject and Parkinson’s patient.

### 2.2. Pre-Processing

**Figure 3:**
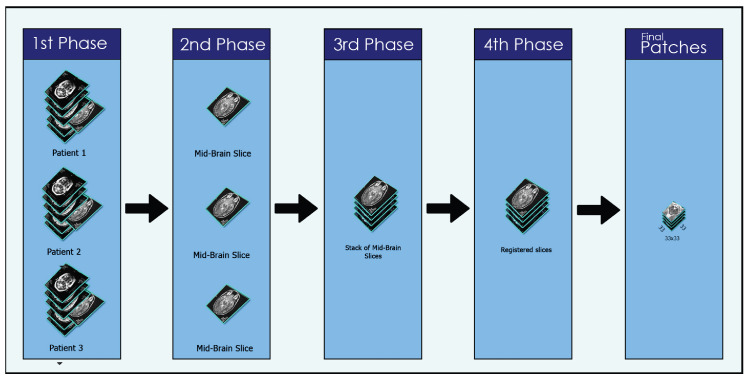
Preprocessing steps.

The MR images were initially stored in the DICOM format and then converted into JPEG using publicly available software known as DICOM to JPEG. Each subject’s data consisted of 45 slices, and only slice number 22 was collected per subject since this slice provides the accurate image of the substantia nigra in PD class. Substantia nigra is a structure in the mid-brain area that controls movement and motor coordination. Dopamine is a substance that is produced in this area and is employed as a signal transmitter. This sends signals about movement and coordination to the brain and other parts of the body. A stack was created by combining slice number 22 from all the subjects. To align the images, intensity-based image registration was carried out using the OpenCV library on the stack. Image registration is the procedure of lining up scans of the brain or other pertinent regions taken from people with Parkinson’s disease. Using image registration techniques, this alignment establishes the spatial relationship between the pictures, enabling a consistent and uniform analysis. By ensuring that the pictures are in a uniform coordinate system, image registration eliminates variances brought on by changes in patient placement or scanning procedures. The primary objective of image registration was to eliminate unwanted and irrelevant information, which could lead our model to learn unnecessary and redundant features. For obtaining a perfect image of substantia nigra, the mid-brain section was cropped using the freehand region of interest (ROI) technique with a window size of 33 × 33. Freehand ROI was used for cropping because the size of the specific organ varies in different patients, and, instead of using fix ROI cropping, the freehand region of interest (ROI) technique provides us better control in cropping the exact position of the organ. This image was the final input to the CNN model. Figure 3 provides a visual representation of the preprocessing steps.

### 2.3. Convolutional Neural Network Architecture

CNN architecture have been widely used for image-relevant tasks, such as image recognition and classification of images, etc. The use of CNNs has effectively improved the performance of many image-relevant tasks. For example, a deep-CNN-based COVID-19 diagnosis system was proposed in [38].The author claimed that the deep-CNN-based dignosis of COVID-19 from sounds like dry cough outperforms other models. In an another article, CNN has been proposed for classification of lung diseases [39]. In this article, the authors applied CNN on chest X-ray images and classified lung diseases into five different disease classes. The results of CNN-based classification were higher than existing methods. The main building blocks in CNN architecture are convolutional layers, activation functions, feature maps, max pooling, and regularization. The CNN architecture begins with convolutional layer that accepts input and uses the convolutional kernels to process the spatial information in local receptive field and report activation value using activation function. The convolutional layers can be stacked over one another, which enables CNN to extract and learn features in increasingly complex hierarchy and provides a features map. The number of generated feature maps depends on the number of convolutional filters used. The activation function encodes the pixel-level spatial neighborhood activation at respective pixel location in feature map. The max pooling layer is after the feature map layer. The main purpose of using the max pooling layer is to reduce the input dimensionality, reduce the risk of overfitting, and reduce computational costs. The result of the max pooling layer can be given to another convolutional layer to create a hierarchical structure. The final feature maps are fully connected to every neuron on dense layer. Finally, softmax function as activation function is also used for classification purpose. Following are the main building blocks of CNN model.

#### 2.3.1. Weights Initialization

Right weight initialization has the key role of deep learning, which reduces the convergence time and brings stability in loss function even after thousands of iterations. Xavier initializer is incorporated in this study that maintains activation variance and back propagation gradient in controlled levels [40,41].

(1) $$Weights∼U\left[-\frac{\sqrt{6}}{\sqrt{w+(w+1)}},\frac{\sqrt{6}}{\sqrt{w+(w+1)}}\right]$$

In Equation (1), *U* is the normal distribution, where *w* is tensor, the weight of input layer, and w+1 is that of output layer.

#### 2.3.2. Convolution of Kernels

After the start of convolution on image, feature maps are generated. Each kernel has a feature map. Feature map *F* can be calculated using the equation below.

(2) $$F=bias+\left[M_{1}*N_{1}+M_{2}*N_{2}+\ldots +M_{n}*N_{n}\right]$$

where *M* shows kernel and *N* shows input channel (2).

#### 2.3.3. Activation Function

Non-linearity into the system is introduced by the activation function. A number of activation functions have been proposed and are still under research. Each activation function has some limitations and is not suitable for every situation: for instance, sigmoid kill gradient. However, ReLU obtained better results when compared with sigmoid and hyperbolic tangent function but suffers from dying ReLU problem. For instance, the large gradient flows through the ReLU update the weights that will never activate at any data point. The other issue with ReLU activation function is that it ignores gradients smaller than zero. LeakyReLU is the improved form of ReLU and tackles the dying ReLU problem by bringing the negative gradient into it. ReLU is defined as

(3) f(x)=max(0,x)

LeakyReLU is defined as

(4) $$f^{\prime }\left(x\right)=f\left(x\right)+\alpha \;\min\left(0,x\right)$$

Here, α is the leakiness parameter, which may be a real number between 0 and 1.

#### 2.3.4. Pooling

Dimensionality of the feature map is reduced by pooling; it makes the system ignore small changes, such as small intensity and illumination change. The prominent pooling layer is max pooling, min pooling, average pooling. The min and max select features with the minimum, maximum value in the pooling kernel, respectively, while the average pooling calculates the average of the features in pooling kernel and returns the average effect of all features. Max pooling is used here in this study, which can be formalized as

(5) $$pool_{k,l}=\max_{p}f^{\prime }{\left(x\right)}_{k+p,l+p}$$

where *k* and *l* correspond to the spatial positions.

#### 2.3.5. Regularization

The main purpose of regulation is to avoid model overfitting. A number of overfitting techniques are available; however, L1 and L2, global average pooling, global max pooling, and batch normalization are well-known among them. Dropout is another effective regulation technique that randomly switches the neurons on and off to learn effectively and contribute in the overall output individually. In this paper, we use Dropout, which removes neurons with probability *p*. The value of the Dropout can be any real value between 0 and 1. The working of Dropout can be observed by the following formula [42]:

(6) $$y_{k}=\sum_{M\;\epsilon \;M^{*}}Pr\left(m\right)y_{k}^{M}$$

where $y_{k}$ is the probable result of the unit *k*, $M^{*}$ is the set of all thinned network, $y^{M}$ is the output of unit *M*, and Pr() shows the probability function.

#### 2.3.6. Fully Connected layers or Dense layers

It is the last layer after convolutional layers. Here, each pixel of the image is considered as neuron and given to each neuron in the fully connected layer. A classifier is used for classification at the end of architecture. Softmax is most common classifier in deep neural networks. It can be defined using Bayes theorem [43].

(7) $$p\left(C_{k}|\;\times \;\right)=\frac{p\left(x|C_{k}\right)\;p\left(C_{k}\right)}{\sum_{j=1}^{n}p\left(x|C_{j}\right)\;p\left(C_{j}\right)}$$

where $C_{k}$ is the targeted class to find, and $C_{j}$ is the *j* = 1,2,3, …, $n^{th}$ class. Its exponential form is as under [43]:

(8) $$\sigma {\left(a\right)}_{k}=\frac{e^{a_{k}}}{\sum_{j=1}^{n}a_{j}}$$

#### 2.3.7. Loss Function

It is used to calculate the compatibility between the given ground truth label and predicted values. The loss function can be custom-designed for a particular task. There are many loss functions based on the nature of the learning problem, but the most common lost function that is used in classification task is categorical cross-entropy. The categorical cross-entropy is used as the cost function. It can be formalized as

(9) $$f_{cost}\left(x\right)=-\sum_{a\;\epsilon \;voxels}\;\sum_{b\;\epsilon \;classes}\;c_{a,b}\;\log\left({\hat{c}}_{a,b}\right)$$

Here, *c* is the actual target class, while $\hat{c}$ is the predicted class in Equation (9).

### 2.4. Proposed Network Architecture

**Figure 4:**
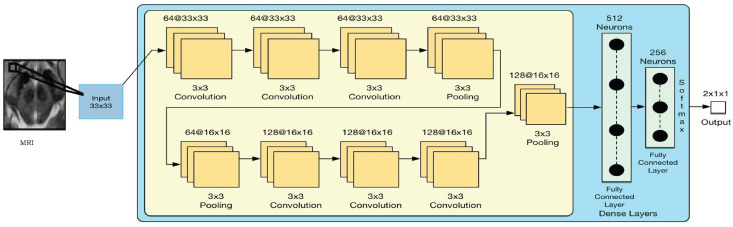
Network architecture.

Our proposed model receives MRI as input and eventually labels it as PD or HC. This method takes advantage of the deeper CNN with a small convolutional kernel of size 3 × 3 throughout the network. The smaller convolutional kernel has fewer parameters to estimate and allows learning and generalizing from limited training data. Conversely, the larger convolutional kernel has larger parameters to estimate, is difficult to generalize, and demands high availability of training data. Each convolutional kernel is followed by advanced activation function (i.e., LeakyReLU). LeakReLU addresses simple ReLU issues, such as dying ReLU by adjusting negative gradients on back propagation. The recently developed batch normalization is used before every LeakyReLU layer to improve the performance. It has the ability to accelerate the training process of the network. The proposed network takes input patch of dimension 33 × 33. The first three convolutional layers are followed by max pooling layer with kernel dimension of 3 × 3 and stride 2 × 2. The output of the first max pooling layer (i.e., feature maps) is 64 (number of channels) 16 × 16 in dimension. The max pooling layer is used to reduce overall dimensionality, which results in fewer learnable parameters. The output feature maps of first max pooling layer are forwarded to next three convolutional layers. The output feature maps of the sixth convolutional layer (i.e., 128 × 16 × 16 in dimension) are forwarded to the second max pooling layer with kernel size of 3 × 3 and stride of 2 × 2. The output feature maps of this pooling layer have dimensions of 128 × 7 × 7. These feature maps are then fully connected to the FC (fully connected) layers. There are two FC layers. The first FC layer has 512 neurons, while the second has 256 neurons. Advanced regularization technique is used, which is dropout with 0.1 value in both FC layers to reduce the risk of network overfitting. At the end of network, softmax layer is used to obtain the classification probabilities. Figure 4 shows graphical representation of the proposed model, while Table 3 shows the architecture along with used parameters of proposed model. In Table 3, “Type” column conv means convolutional layer and Max-pool means max pooling layer. In “Inputs” column, the first value is the number of input channels and next two values are the dimension of the feature map or patch size.

## 3. Results

This section discusses the outcome performance of the network on the Parkinson’s dataset.

### 3.1. Performance Measures

Area under curve (AUC), classification accuracy, sensitivity, and specificity are used to evaluate the performance of the proposed model. These are calculated as in Equations (10)–(12).

(10) $$ACC=\frac{TP+TN}{TP+FP+FN+TN}\times 100\%$$

(11) $$Sensitivity=\frac{TP}{TP+FN}\times 100\%$$

(12) $$Specificity=\frac{TN}{FP+TN}\times 100\%$$

### 3.2. Experimental Setup

The NVIDIA Geforce 940MX GPU, which supports Keras, has been utilized to run the CNN. The Keras Python deep learning API enables the usage of both Theano and Tensorflow. The Theano library and Sequential model are used as a CNN model.

### 3.3. Experiments

In order to evaluate the robustness of the network, a number of experiments have been performed. The proposed model has been applied to the patches as well as to the complete image. Different model parameters have been tested to obtain a robust model. Furthermore, several times, the model has been executed to find the performance validity of the proposed model. The accuracy of the proposed model has been recorded after each epoch. The system is evaluated on the training set as well as on the test set. Four of the experiments are elaborated below. Experiments 1 and 2 show the highest and the lowest accuracy, respectively, archived during training and validation with the same input and network settings. In experiment 3, the last convolutional layer is eliminated in order to reduce the computational cost while keeping the input the same as in experiments 1 and 2. However, in experiment 4, the same networking settings are maintained. The network is tested on the full mid-brain as input rather than ROI patches.

### 3.4. First Experiment

**Figure 5:**
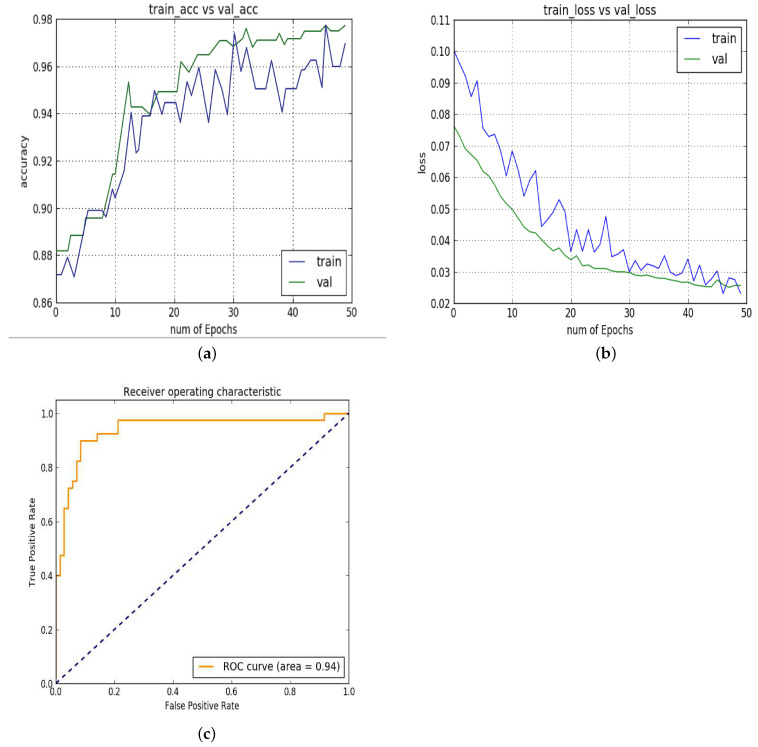
Experiment 1: (**a**) training vs. validation accuracy; (**b**) training vs. loss; (**c**) ROC.

In experiment 1, Figure 5a shows the training and testing accuracy. The x-axis shows the number of epochs, while the Y-axis shows accuracy. The line in green color is for validation accuracy, while blue is for training accuracy. The accuracy increases with the number of epochs. The validation accuracy reached up to 98%. Figure 5b shows the training loss vs. validation loss. The × axis represents the numbers of epochs and the y-axis represents the loss. Figure 5c shows the ROC curve for the proposed architecture on the test set. The x-axis represents the false positive rate and the y-axis represents the true positive rate. In this experiment, the proposed model obtained an AUC of 0.94 on the test set.

### 3.5. Second Experiment

**Figure 6:**
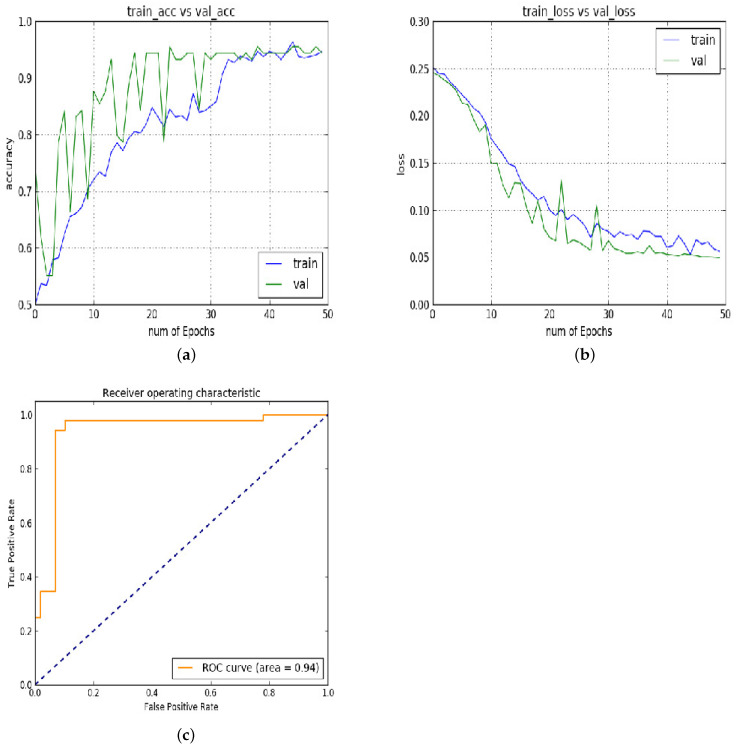
Experiment 2: (**a**) training vs. validation accuracy; (**b**) training vs. validation loss; (**c**) ROC.

In experiment 2, Figure 6a–c show the results of the same model repeated for 50 epochs on the same input patches for validating the performance of the model. In this experiment, the training and validation accuracy decreased to 95%. This is the least accuracy obtained by the proposed model. The 3% decrement in the accuracy is due to the noise in input.

### 3.6. Third Experiment

**Figure 7:**
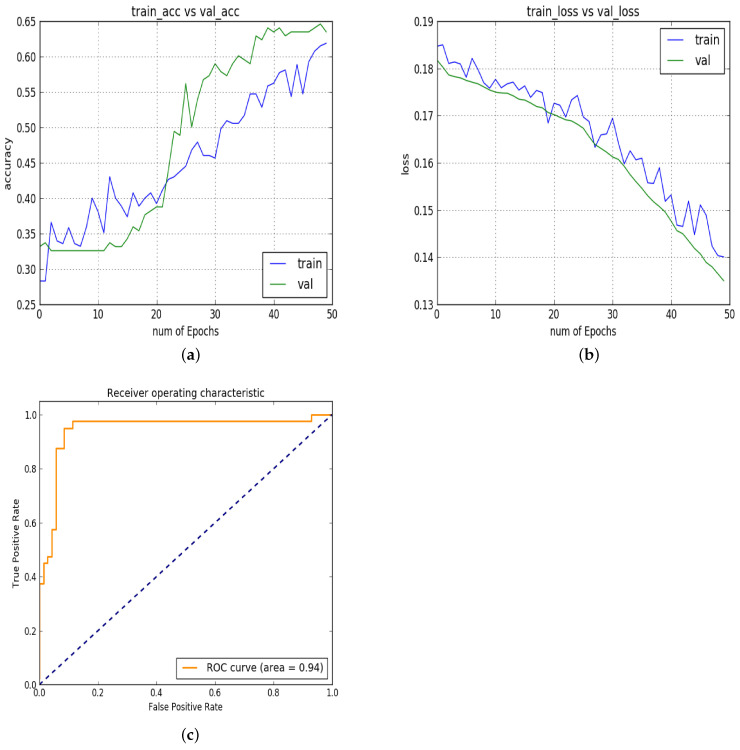
Experiment 3: (**a**) training vs. validation accuracy; (**b**) training vs. validation loss; (**c**) ROC.

In experiment 3, Figure 7a–c show the results of the third experiment in which the last convolutional layer is eliminated. This last layer is removed to reduce the computational cost, but the accuracy of the model is greatly affected. The AUC remains constant on the test set, but the accuracy is reduced to 65% on validation as well as on testing. The AUC remains the same up to 94% on the test set.

### 3.7. Fourth Experiment

**Figure 8:**
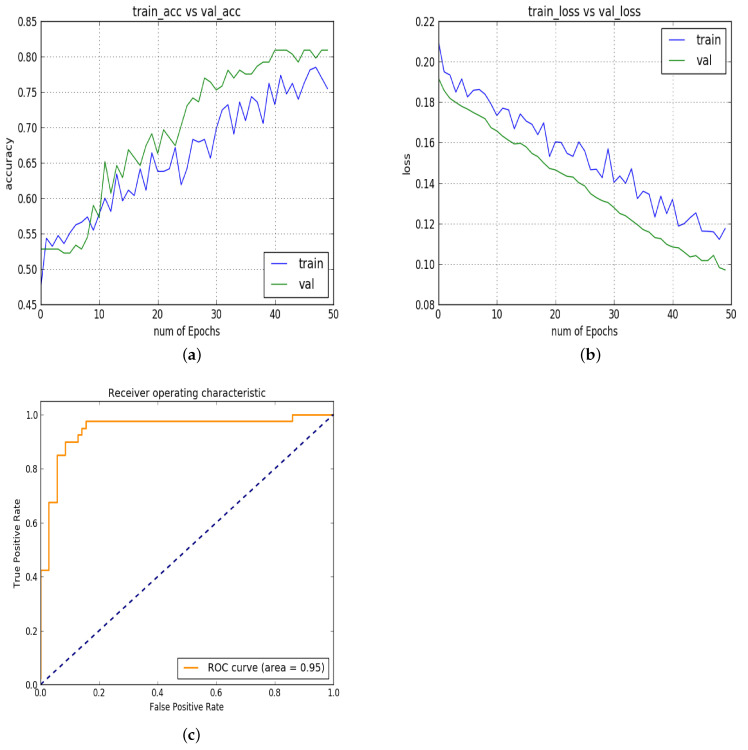
Experiment 4: (**a**) training vs. validation accuracy; (**b**) training vs. validation loss; (**c**) ROC.

In experiment 4, Figure 8a–c show the results of the next experiment in which the model is applied to a full slice of MRI instead of patches. The AUC is constant, but the accuracy is reduced to 85%. The comparison of the several experiments shows that the proposed architecture performed better with patches and produced high AUC and accuracy on validation and test sets.

## 4. Discussion

Numerous experiments have employed various network setups. The network parameters include layer count, input size, and other network features. The accuracy has consistently ranged from 94 to 98 percent. In Table 4, the AUC, sensitivity, specificity, and average classification accuracy have been shown. Also, we have generated the confusion matrix representing the true and false classification in Table 5.

Detection of PD from MR imaging cannot be considered a novel task since many researchers have attempted to classify PD and HC. Table 1 represents a comprehensive analysis of the prior work. In these studies, different groups of ML techniques are applied that contain supervised models, such as SVM [17,24,27,30,32,44], and unsupervised models, like [28,33]. These models achieved promising results, but their accuracies are more likely variable accuracies. In many of the mentioned works, the authors used millions of features of single or multiple modalities with limited datasets using SVM, which creates a hyperplane in the n-feature dimensional space. Using this strategy can achieve high accuracy, but it has a chance of overfitting.

To compare the results, with Alexnet [22] being the pioneers to use a novel strategy of using ROI base patches, when it comes to the biological domain, PD is associated with the substantia nigra. There is a chance of high structural changes in this organ as compared to the rest of the brain. Providing the input of the specific image (substantia nigra) to the network rather than the full image is the key factor of achieving promising results. The performance comparison of our model with other classifiers can be seen in Table 6. The results show that the proposed model suppresses the previous models, while experiment 4 confirms the involvement of mid-brain (patches) in PD classification.

## 5. Conclusions

In conclusion, this paper proposes a customized CAD system that utilizes convolutional neural networks to accurately classify MRI patches into Parkinson’s and healthy patterns. The model successfully extracts and learns the patterns from the training samples of the benchmark PPMI dataset, resulting in improved results. The findings demonstrate that the proposed model can autonomously learn accurate features of Parkinson’s disease. However, the study highlights the challenge of overfitting in working with a limited dataset. Nevertheless, the proper design and integration of the dropout layer in the model enable effective suppression of the overfitting problem. Overall, the proposed CNN-based model offers a promising approach for the automatic and precise classification of Parkinson’s disease, and it has the potential to benefit clinical practice in the future.

### 5.1. Contribution

With the increasing trend of computer-aided diagnosis, it has become feasible to avail these technologies for diagnosing complex diseases. Despite limited resources, these technologies are being used along with machine learning approaches for diagnosis of different diseases in many biomedical research labs. A computer-aided diagnosis based on convolutional neural network is presented in this paper. The performance of the model has been analyzed in detail on the basis of accuracy, sensitivity, specificity, and AUC. One of the main objectives of the proposed system is to reduce the incorrect diagnosis of PD and to detect the disease in early stages to improve the QoF of patients. To the best of our knowledge, this is the very first attempt to apply convolutional neural network on ROI for classification of Parkinson’s disease. Although there is no cure for the disease itself, there are treatments available that help in reducing the symptoms for newly diagnosed patients. This maintains QoF for as long as possible.

### 5.2. Future Work

The proposed network is more simple, with fewer feature maps and layers. The complex features can be learned by complex organization of the network; however, a complex network requires a huge amount data. In the future, it is intended to work on the problem as the dataset is also updating with new patient records. The efforts will continue to improve the correct diagnosis of Parkinson’s disease.

## Figures and Tables

**Table 1:** Summary of literature review results.

Reference	Data Type	Number of Subjects	Methods Used	Accuracy	Year
[24]	MRI Scans	PD (n = 28)	Voxel-based morphometry	PD vs. HC: 83.2	2014
		HC (n = 28)	Principal component analysis	PSP vs. PD: 84.7	
		PSP (n = 28)	Support vector machine	PSP vs. HC: 86.2	
[27]	MRI Scans	Tremor dominant PD (n = 15)	Voxel-based morphometry	100	2014
		ET with rest tremor (n = 15)	Diffusion tensor imaging		
			Support vector machine		
[28]	MRI Scans	PD (n = 518)	Self-organizing maps	99.9	2015
		HC (n = 245)	Support vector machine		
		SWEDD (n = 68)			
[29]	MRI Scans	PD (n = 30)	Region-of-interest-based	86.67	2015
		HC (n = 30)	Support vector machine		
[30]	MRI Scans	PD (n = 204)	Volumetry	80	2016
		MSA-C (n = 21)	Support vector machine		
		PSP-RS (n = 106)			
		MSA-P (n = 60)			
[31]	MRI Scans	PPMI cohort	Joint feature-sample selection	81.9	2016
		HC (n = 169)			
		PD (n = 374)			
[32]	MRI Scans	HC (n = 38)	Functional connectome	80	2017
		PD (n = 27)	Support vector machine		
[17]	MRI Scans	HC (n = 169)	Connectivity measures	93	2018
		PD (n = 374)	Support vector machine		
[33]	MRI Scans	PD (n = 26)	Voxel-based morphometry	PD vs. MSA: 96	2018
		HC (n = 26)	T2 relaxometry, DTI		
		MSA-P (n = 16)	Self-organizing maps		
		MSA-C (n = 13)			
[34]	MRI Scans	HC (n = 39)	NM-MRI-based atlas of Substantia nigra	79.9	2019
		PD (n = 40)			
[16]	MRI Scans	HC (n = 35)	NM-MRI-based atlas of Substantia nigra	89	2019
		PD (n = 25)			
[22]	MRI Scans	PPMI	CNN model AlexNet	88.9	2019
		HC = 82	Transfer learning		
		PD = 100			

**Table 2:** Details of subjects.

	Total	Male	Female	Age (Years)
**PD**	250	173	77	60 ± 10
**HC**	250	136	114	60 ± 10

**Table 3:** Detailed structure of CNN architecture. Conv. is used for convolutional layers, Max-Pool. is used for max pooling layers, and FC is used for fully connected layers.

Layer No.	Type	Filter Size	Stride	# Filters	FC Units	Input
**Layer 1**	Conv.	3 × 3	1 × 1	64	-	33 × 33
**Layer 2**	Conv.	3 × 3	1 × 1	64	-	64 × 33 × 33
**Layer 3**	Conv.	3 × 3	1 × 1	64	-	64 × 33 × 33
**Layer 4**	Maxpool.	3 × 3	2 × 2	-	-	64 × 33 × 33
**Layer 5**	Conv.	3 × 3	1 × 1	128	-	64 × 16 × 16
**Layer 6**	Conv.	3 × 3	1 × 1	128	-	128 × 16 × 16
**Layer 7**	Conv.	3 × 3	1 × 1	128	-	128 × 16 × 16
**Layer 8**	Maxpool.	3 × 3	2 × 2	-	-	128 × 16 × 16
**Layer 9**	FC	-	-	-	512	6272
**Layer 10**	FC	-	-	-	256	512
**Layer 11**	FC	-	-	-	2	256

**Table 4:** Classification results.

Accuracy	Specificity	Sensitivity	AUC
96 ± 2	96.87 ± 3.13	95.83 ± 0	94.5 ± 0.5

**Table 5:** Confusion matrix.

		Predicted	
		**Yes**	**No**
**Actual**	Yes	49	1
	No	3	47

**Table 6:** Performance comparison.

Method	Accuracy	Sensitivity	Specificity	AUC
PDF, PCA, SVM [45]	73.1	67.5	78.7	-
Complex network, SVM [17]	88	85	88	-
CNN(AlexNet), Transfer learning [22]	88.9	89.3	88.4	-
SVM [46]	92.3	90	94	97
GA-ELM [47]	89.22	92.35	92.35	-
SVM [44]	86.67	-	-	-
**ResNet50**	82	83.5	85	85.4
**Proposed CNN Model**	**96**	**96.8**	**95.8**	**95**

## Data Availability

The data will be made available on request.
